# Draft Genome Sequence of Lactobacillus helveticus Strain Lh 12 Isolated from Natural Whey Starter

**DOI:** 10.1128/genomeA.00139-18

**Published:** 2018-03-08

**Authors:** Gaia Bertani, Daniela Bassi, Monica Gatti, Pier Sandro Cocconcelli, Erasmo Neviani

**Affiliations:** a Department of Food and Drug, University of Parma, Parma, Italy; b Institute of Microbiology, Università Cattolica del Sacro Cuore, Cremona, Italy

## Abstract

Lactobacillus helveticus is a lactic acid bacterium widely used in cheese-making and for the production of bioactive peptides from milk proteins. Here, we describe the draft genome sequence and annotation of L. helveticus strain Lh 12 isolated from natural whey starter used in the production of Grana Padano cheese.

## GENOME ANNOUNCEMENT

Lactobacillus helveticus is a homofermentative, thermophilic lactic acid bacterium widely used as a starter culture in the manufacture of Swiss-type and long-ripened Italian hard-cooked cheeses and as a flavor-enhancing adjunct culture for other types of cheese (1). Furthermore, this microorganism is able to produce peptides with biological functions, such as inhibitory activity on the angiotensin-converting enzyme (2).

L. helveticus Lh 12 was originally isolated from natural whey starter (NWS), the undefined starter culture used to produce Grana Padano, a protected designation of origin cheese. Using a technique known as back-slopping, NWS is obtained from the previous day’s cheese-making whey and is incubated at a decreasing temperature. This method produces a large number of thermophilic lactic acid bacteria that, together with the raw milk microbiota, preserve the final product from damage and determine the final attributes of the cheese (3, 4).

Differences in the biotype composition of NWS, which distinguish natural from selected cultures, are modulated by the incubation conditions of the drained whey, in strict synergy with small variations in the applied dairy technology (3, 5). Nowadays, the interest in these undefined complex starter cultures is growing because of their low vulnerability to bacteriophage attack and because they represent a source for the isolation of new dairy strains with interesting functional characteristics (6). In a previous study, different strains of L. helveticus were analyzed for genes involved in the proteolytic system and amino acid catabolism (1). Strain Lh 12 (named UPR2 in reference 1) was genetically different from the other strains, and therefore, sequencing of this genome may reveal alternative and interesting metabolic pathways.

The genome was sequenced with a 917.3-fold overall genome coverage using an Illumina HiSeq 1000 platform from the Parco Tecnologico Padano (PTP) Genomics Platform (Lodi, Italy). The obtained reads were *de novo* assembled using SPAdes software version 3.1.0. This strategy resulted in 334 contigs with a calculated genome size of 2.13 Mb and a GC content of 36.6%. A total of 2,327 genes were predicted by annotating the genome with both the NCBI Prokaryotic Genome Automatic Annotation Pipeline (PGAP) and the Rapid Annotations using Subsystems Technology (RAST) annotation server (7, 8). The draft genome contained 2,327 genes in total, including 1,820 coding genes, 5 rRNAs, 61 tRNAs, and 502 hypothetical proteins. The functional annotation was performed using RAST version 2.0. The proteolytic nature of this strain was reflected in the genome by the presence of genes involved in amino acid metabolism. Strain Lh 12 also harbors genes involved in sugar metabolism, transport, and uptake, as well as genes participating in lactose, galactose, fructose, chitin, and *N*-acetylglucosamine utilization.

The genome analysis also revealed genes involved in stress response (heat shock response and osmotic and oxidative stress response) and in DNA metabolism (coding for clustered regularly interspaced short palindromic repeat-associated proteins and restriction-modification systems). Finally, two intact and three partial phage regions were identified by using the PHAge Search Tool (PHAST) (9).

### Accession number(s).

This whole-genome shotgun project has been deposited in DDBJ/ENA/GenBank under the accession number LSVI00000000. The version described in this paper is the first version, LSVI01000000.
